# The Impact of Energy Drinks on the Genesis of Cardiac Arrhythmias: A Narrative Review

**DOI:** 10.7759/cureus.117819

**Published:** 2026-10-12

**Authors:** Kelsey Andux, Harvey N Mayrovitz

**Affiliations:** 1 Dr. Kiran C. Patel College of Osteopathic Medicine, Nova Southeastern University, Fort Lauderdale, USA; 2 Dr. Kiran C. Patel College of Allopathic Medicine, Nova Southeastern University, Fort Lauderdale, USA

**Keywords:** alcohol, arrhythmia, caffeine, energy drinks, exercise, ginseng, healthy individuals, lifestyle, qtc interval, taurine

## Abstract

This narrative review aims to synthesize research on energy drink use and the development of arrhythmias among healthy individuals without preexisting conditions. A special focus is given to the electrocardiogram (EKG) following beverage consumption. A literature search was conducted on the Web of Science, Embase, Medline, and Cochrane databases for original, peer-reviewed studies that reported EKG findings following energy drink consumption. Secondary research and studies that had subjects with preexisting cardiac conditions were excluded. Ten controlled experimental studies and seven case reports met the inclusion criteria. Currently available evidence from controlled experimental studies involved one-time, one-day intake of energy drinks. Beverages varied in volume, content, and consumption rates across studies. Some acute EKG changes, including prolongation of the corrected QT (QTc) interval, have been reported in prospective studies in relation to energy drinks. However, no clinically significant arrhythmias were recorded. Although the studies in this review suggest that limited use of energy drinks is unlikely to cause an arrhythmia in healthy individuals, their arrhythmogenic potential cannot be ruled out. Case reports related to otherwise healthy individuals demonstrate a potential relationship between energy drink usage and the development of atrial fibrillation, especially with the co-ingestion of alcohol. The presence of confounding factors in these case reports makes it unclear whether energy drinks alone, or with the presence of additional triggers, may precipitate an arrhythmia. Future research should focus on energy drink use over time and on the contribution of lifestyle factors to better assess the impact of energy drinks on arrhythmia genesis.

## Introduction and background

Since their introduction in the 1960s, energy drinks have become one of the fastest-growing beverages [1,2]. They are generally marketed towards young adults, with claims of increased stamina, wakefulness, and attention upon intake [2,3]. Current trends indicate that young adults aged 18-34 consume energy drinks one to four times a month [2]. Moreover, one in three teenagers aged 12-17 reports consuming energy drinks regularly [3]. These beverages contain caffeine and additional ingredients, such as guarana, taurine, B vitamins, ginseng, and L-carnitine [3,4]. An increase in intracellular sodium and calcium levels causes a positive ionotropic effect when taurine is administered acutely [3]. Studies indicate a link between ginseng and electrophysiologic abnormalities, such as atrial fibrillation (AFib) and long QT syndrome [3]. Additionally, caffeine is known to increase sympathetic tone and peripheral vascular resistance in some people [3]. Clinical studies have demonstrated a relationship between energy drinks and acute elevations in blood pressure and heart rate in adults, likely due to caffeine [3,5]. According to the Food and Drug Administration (FDA), the maximum safe dose of caffeine for a soft drink is 71 mg per 12 oz [3,6]. In a single beverage, energy drinks can exceed this upper limit up to three times [3]. In general, caffeine intake of up to 400 mg/day is not associated with adverse cardiovascular effects in healthy adults [7]. However, current evidence suggests that, at high doses, caffeine leads to AFib and premature ventricular contractions [8].

There have been reports that document sudden cardiac death, ventricular fibrillation, and coronary vasospasm secondary to energy drink consumption [9]. From 2007 to 2011, the number of emergency room visits related to energy drinks doubled, reaching over 20,000 [10]. Despite the popularity of energy drinks and related reports of adverse cardiovascular events, the relationship between energy drink ingestion and cardiac arrhythmias is not entirely understood. Therefore, this review aims to examine the existing literature on energy drinks and to describe and consolidate their use and its potential to cause arrhythmias in otherwise healthy individuals.

## Review

Study design

A narrative review approach was chosen over a systematic review due to heterogeneity in study design, participant population, energy drink composition and dose, and electrocardiogram (EKG) endpoints. Due to the narrative approach, no statistical pooling, quantitative synthesis, meta-analysis, or risk-of-bias assessment were performed. 

Search strategy

A comprehensive literature search was conducted in November 2025 using Web of Science, Embase, Medline, and Cochrane databases. Articles were screened and selected independently by one reviewer. The search strategy used the following combination of terms ("energy drinks" OR "performance drink" OR "energy beverage" OR "caffeinated drink" OR "stimulate drink" OR "stimulant beverage") AND ("arrhythmia" OR "irregular heartbeat" OR "abnormal heart rhythm" OR "irregular cardiac rhythm" OR "ectopic beats" OR "skipped beats"). Peer-reviewed original research studies were eligible if they included EKG results after energy drink consumption and were published in English from January 2000 to November 2025. An additional supplemental search was conducted in March 2026. Exclusion criteria for both searches included literature reviews, animal studies, conference abstracts, and studies in which patients had a pre-existing cardiovascular condition. Therefore, individual case reports, within a case series, involving those with pre-existing cardiac conditions were excluded in this review. Ten prospective studies and seven case reports were included in this review and are discussed separately in their individual sections.

Main findings from prospective studies

Electrocardiographic Effects

Through multiple studies, the arrhythmia-inducing effects of energy drinks have been assessed by examining EKG changes following ingestion of various energy drink doses, primarily in healthy young subjects. Although some changes in various EKG intervals have been described, they have been small, and the incidence of malignant arrhythmias, though reported in individual case studies, has been relatively rare in prospective studies. Table 1 summarizes the key aspects of these 10 studies.

**Table 1:** Summary of study findings included in this review Abbreviations: ED = energy drink; EKG = electrocardiogram; QTc = corrected QT interval; HR = heart rate; BP = blood pressure; SBP = systolic blood pressure; DBP = diastolic blood pressure; Tp-e = T wave peak to end; ER = emergency room; FMD = flow mediated dilation; VO2 = maximum oxygen consumption

Reference	Study Design	Sample Size	Intervention/Concept	Energy Drink Main Components	Outcomes Measured	Key Findings	Limitations
Shah et al., 2016 [11]	Randomized, double-blind, crossover study	27	Cardiovascular effects of consuming high-dose EDs and Panax Ginseng	Group 1: 32 oz ED containing 4000 mg taurine, 800 mg ginseng, 320 mg caffeine; Group 2: 800 mg of Panax ginseng in 70 mL cherry syrup, 20 mL lime juice, and 410 mL carbonated water; Group 3: Placebo containing 70 mL of cherry syrup, 20 mL lime juice, and 410 mL carbonated water	EKG and BP	Two hours after ED intake, QTc interval increased, and PR interval decreased. No changes in QT or QRS intervals.	Drinking 32 oz of an ED in 45 minutes is not representative of consumer patterns.
Fletcher et al., 2017 [12]	Randomized, double-blind, controlled, crossover study	18	Examine cardiovascular safety of high ED consumption	Group 1: 32 oz ED containing 320 mg caffeine, 108 g sugar, B vitamins, taurine, Panax ginseng, L -carnitine, glucuronolactone, inositol, guarana extract, and maltodextrin; Group 2: 32 oz placebo containing 320 mg caffeine, 40 mL lime juice, 140 mL cherry syrup, and carbonated water	EKG, BP, HR, adverse effects	Small, statistically significant increase in QTc interval > 10 ms from baseline in the ED group only at two hours after intake.	Food intake was not monitored.
Elitok et al., 2015 [13]	Prospective, open-label study	50	"RedBull" effects on ventricular repolarization	355 mL "Redbull" containing 114 mg caffeine, 1.42 mg taurine, 84.2 mg glucuronolactone, 39.1 g sucrose and glucose	EKG, Tp-e interval, Tp-e/QT ratio	Significant changes were found in HR, SBP, and DBP after ED consumption. No significant changes in EKG parameters, Tp-e interval, or Tp-e/QT ratio.	Low-dose ED.
Wiklund et al., 2009 [14]	Crossover study	10	Examine if ED alone and combined with alcohol before exercise	750 mL "Red Bull" containing 3000 mg of taurine, 240 mg of caffeine, 0.4 g of ethanol/kg body weight	EKG, heart rate recovery, heart rate variability	No subject developed a clinically significant arrhythmia after drinking the ED.	Small sample size.
Mandilaras et al., 2022 [15]	Prospective, randomized, double-blind, placebo-controlled, crossover clinical study	26	Investigate acute cardiovascular effects of ED in the pediatric population	Group 1: ED containing 0.32 mg/mL caffeine, 2 mg/mL taurine, 2.4 mg/mL glucuronolactone, 0.1 mg/mL ginseng aroma extract, 0.1mg/mL guarana extract, B vitamins; Group 2: Placebo containing fruit juice, water, water, fruit and vegetable extracts	HR and EKG	Supraventricular premature contractions significantly increased with ED consumption. There were no significant changes in QTc. At two hours, the ED group had a significantly lower QT-RR relationship than the placebo group.	Small sample size; some participants were able to tell what treatment they were receiving.
Higgins et al., 2020 [16]	Observational open-label study	44	ED consumption and endothelial function	24 oz "Monster Energy" containing 240 mg caffeine, 54 g sugar, 360 mg sodium, 2,000 mg taurine, 400 mg ginseng extract, glucuronolactone, guarana extract, L-carnitine	FMD, BP, HR, EKG	No significant EKG changes with ED consumption. BP and HR significantly increased after 15 min. FMD at the brachial artery decreased 90 min after ED.	Low-dose ED.
Özde et al., 2020 [17]	Prospective, observational and open-label study	54	Red Bull consumption and atrial conduction time delay	330 mL "Red Bull" containing 114 mg caffeine, 1.42 mg taurine, 84.2 mg glucuronolactone, 39.1 g sucrose and glucose	Atrial electromechanical coupling	Electromechanical conduction time increased at the lateral and septal mitral annuli two hours after ED intake.	Food intake was not standardized.
Busuttil & Willoughby, 2016 [18]	Questionnaire	60	Relationship between ED consumption and ER visits	N/A	ED habits, BP, EKG	Of the 60 patients, 15 had consumed at least one ED 24 hours before admission to the emergency department. There were no significant EKG changes between ED consumers and non-consumers. No association was found between ED usage and other cardiac symptoms.	Small sample size.
Sillivent et al., 2012 [19]	Randomized double-blind, controlled study	7	Cardiovascular effects of the ED, "Redline" consumption before and after vigorous exercise	Group 1: Placebo 240 ml Wild Berry Crystal Light; Group 2: "Redline" containing 240 mg caffeine, potassium, vitamin C, beta-alanine, ascorbic acid	HR, BP, VO2 max	No statistically significant effects; however, the Redline group had five times as many ectopic beats (106) as the placebo group (21).	Small sample size.
Kozik et al., 2016 [20]	Prospective observational study	14	EDs effects on BP, electrolytes, EKG readings, and active bleeding time	32 oz "Monster Energy" containing 320 mg caffeine, sugar, taurine, ginseng extract, glucuronolactone, guarana extract, L-carnitine	BP, HR, EKG, magnesium, calcium, potassium, activated bleeding time	SBP, QTc interval, magnesium and calcium increased from baseline. QTc interval increased when EDs were combined with light exercise.	Small sample size.

The corrected QT interval (QTc) was the most widely studied EKG parameter. In a randomized, double-blind crossover study, the effects of energy drinks on EKG findings were evaluated in 27 healthy participants [11]. They were given a placebo drink, 32 oz of an energy drink, and an 800 mg Panax Ginseng drink, with a six-day washout period between treatments. Participants consumed each beverage within 45 minutes and were evaluated at 1, 2, 3.5, and 5.5 hours post consumption. Results showed a small but statistically significant increase in the QTc interval compared with the placebo drink, observed only at the two-hour assessment. The increase was not present compared to drinking the Ginseng sample. The QTc difference was reported as 3.37 ± 10.7 ms; however, changes from baseline QTc values were not reported, making interpretation of these findings difficult. A similarly designed study was conducted in which 18 healthy subjects, on one occasion, consumed an energy drink and, on another, a caffeinated placebo drink [12]. Assessments were made at 2, 4, 6, and 24 hours post consumption. The reported baseline QTc for the full group was 413 ± 17.3 ms. Two hours after drinking the caffeine drink, QTc decreased by 10.4 ± 14.8 ms, but increased by 0.44 ± 18.4 ms two hours after drinking the energy drink. Although the researchers reported this as a significant difference in responses, their statistical analysis was inconclusive; if anything, it was driven more by the decrease in the caffeine response than by the increase attributed to the energy drink. Further, only at the two-hour mark was any difference observed between the caffeine and energy drinks. The small QTc changes observed in these two studies are inconsistent with a substantial impact of these specific energy drinks on QTc.

Another study utilized a lower-dose 12-oz energy drink, "Red Bull," containing 115 mg of caffeine, to examine its effects on the time difference between the peak of the T-wave and its end (Tp-e interval), which is proposed as a superior marker for arrhythmia development following energy drink consumption [13]. In the study, no significant differences were observed in the PR, QTc, QT, or Tp-e intervals compared with baseline. A 2009 study utilizing high-dose energy drinks (three cans of "RedBull") observed no change in QTc intervals in 10 young adult subjects, nor did they develop any clinically significant arrhythmia [14].

Because energy drinks are sometimes consumed by children, information on their impacts in children was the focus of a study that examined the effects of energy drinks versus a placebo drink on the cardiovascular system in 26 healthy children and teenagers, with an average age of 14.49 years. Following the European Food Safety Authority, participants were given an energy drink containing the maximum daily dose of caffeine for minors (3 mg caffeine per kilogram body weight) and a placebo drink with no caffeine. The participants ingested one of these on each of two consecutive days. Each day, they were evaluated at one, two, three, and four hours after ingesting each drink. Results showed no significant changes in the QTc interval between treatments, with all post-QTc values ranging from 427 ± 25 ms to 434 ± 25.6 ms, whether drinking the placebo or the energy drink [15]. This finding is consistent with the previously described adult low or no effects on QTc. However, some supraventricular extrasystoles were recorded after energy drink consumption that were not present with the placebo; no malignant ventricular arrhythmias or supraventricular tachycardias were recorded during the study.

A 2021 study examined the effects of "Monster Energy," a different energy drink, on the QTc interval among 44 medical students. Participants drank 24 oz of the beverage, which contains 240 mg of caffeine in addition to other ingredients [16]. No significant effects on QTc were observed. In this study, evaluations conducted 90 minutes after drinking appeared to reveal a deficit in brachial artery flow-mediated dilation, with dilation decreasing from about 5% to 2.5%. It is unknown whether such vascular effects contribute to EKG changes.

Atrial Electromechanical Coupling

AFib has been stated as a potential adverse cardiovascular event related to energy drinks [11,12,17]. A possible cause was sought by assessing the inter- and intra-atrial electromechanical delay associated with energy drinks. A prolongation of this delay, between the onset of the EKG P-wave and atrial contraction, would be consistent with contributing to the triggering of AFib. A single can of the energy drink "Red Bull" was ingested by 54 young healthy adults, and the response was evaluated two hours later [17]. Systolic blood pressure, heart rate, and the atrial electromechanical delay were significantly increased compared to baseline (p < 0.001). The pre-drink lateral delay increased from 49.7 ± 11.2 ms to 54.1 ± 11.0 ms. It was concluded that increased delay may contribute to energy drink-induced AFib, although no AFib episodes were observed in this study. 

Confounding Factors

Co-ingestion of energy drinks with alcohol has been found to exacerbate palpitation frequency [18]. Although not statistically significant, when given an energy drink before physical activity, ectopic beats were found to increase fivefold during exercise [19]. Furthermore, a 2016 study evaluating exercise and energy drink use recorded elongation of the QTc interval over 500 ms after 30 seconds of light activity [20]. QTc values normalized once heart rates returned to baseline.

Case report findings

As shown in Table 2, a total of seven case reports, including 10 subjects, were included in this review. All subjects were male. AFib was the most common arrhythmia reported, with subjects returning to sinus rhythm both spontaneously and with pharmacologic intervention [21-25]. Two of the subjects were reported to have entered ventricular fibrillation and required defibrillation to restore sinus rhythm [26,27]. All subjects reported being symptom-free, with no recurrence of arrhythmia at their follow-up appointments. Palpitations were the most frequently reported symptom at the emergency room, representing eight subjects [21-24,27]. Anxiety and shortness of breath were other common presentations [21,24,25]. A total of four cases involved co-ingestion with alcohol.

**Table 2:** Summary of case reports included in this review Abbreviations: yo = year old; SOB = shortness of breath; ED = energy drink; AFib = atrial fibrillation; BPM = beats per minute; EKG = electrocardiogram; VF = ventricular fibrillation; ICD = implantable cardioverter-defibrillator; D/C = direct current; CPR = cardiopulmonary resuscitation

Citation	Age/Gender	Presentation	Energy Drink and Co-ingestions	Electrocardiogram Abnormalities	Interventions and Patient Outcome
Zorlu, 2020 [21]	23 yo male	SOB, palpitations, anxiety	750 mL of ED two hours before admission	AFib, rate of 162 bpm	Treated with propafenone. Returned to normal sinus rhythm
Sattari et al., 2016 [22]	28 yo male	Bloody emesis and palpitations	Two cans of "Monster Energy" (320 mg caffeine) daily for several months, with the last ED one day before admission. Two to three beers	AFib, rate of 130 bpm	Diltiazem and metoprolol given at the hospital. EKG normal and arrhythmia free at 6 and 12 month follow-up
Di Rocco et al., 2011 [23]	Case 1: 14 yo male Case 2: 16 yo male	Case 1: Palpitations after running; Case 2: Intoxication and vomiting after head trauma from a fall	Case 1: Unknown amount of a caffeinated beverage the day before admission; Case 2: Unknown volume of "Red Bull" mixed with Vodka	Case 1: AFib, rate of 130 bpm; Case 2: AFib, rate of 160 bpm	Case 1: Digoxin restored sinus rhythm. No EKG or cardiac abnormalities at 1 month follow-up; Case 2: Normal saline with spontaneous restoration to sinus rhythm. Normal sinus at 1 week follow-up
Mattioli et al., 2018 [24]	Case 1: 22 yo male, Case 2: 23 yo male, Case 3: 26 yo male	Case 1: Palpitations, anxiety, nausea; Case 2: Palpitations and anxiety; Case 3: Anxiety, nausea, precordial discomfort with palpitations	Case 1: 750 mL of ED three hours before admission; Case 2: 600 mL of ED one hour prior to symptoms; Case 3: 600 mL of ED and 30 g of alcohol two hours before admission	Case 1: AFib, rate of 135 bpm; Case 2: AFib, rate of 150 bpm; Case 3: AFib, rate of 170 bpm	Case 1: Propafenone restored sinus rhythm. Denied reoccurrences at 12 months; Case 2: Spontaneous conversion to sinus rhythm. Negative at 12 months; Case 3: Converted to sinus after saline infusion. Negative at 12 months
Hanif et al., 2020 [25]	22 yo male	SOB, restless, vomiting	Two cans of ED, total of 222 mg caffeine, on the same day of admission	AFib with a ventricular rate of 150 bpm	Spontaneous resolution with normal EKG findings at one month
Enriquez & Frankel, 2017 [26]	19 yo male	Cardiac arrest, pulseless, apneic	Three 8 oz "Monster Energy" within two hours	VF	Defibrillation restored sinus rhythm. Implantation of subcutaneous ICD
Osman et al., 2019 [27]	32 yo male	Palpitations, lack of sleep, cardiac arrest after initial assessment	48 cans of 250 mL ED mixed with vodka over three days	VF	Biphasic D/C shock and CPR restored sinus rhythm. Remained asymptomatic after discharge

Discussion

This narrative review examines the role of energy drinks as a potential factor in the development of cardiac arrhythmias among healthy individuals. The literature on this topic falls into two broad categories: controlled experimental studies and case studies. All experimental studies that met our inclusion criteria investigated EKG changes after participants drank an energy drink. These investigations placed particular emphasis on the QTc interval but also reported any EKG irregularities, if present. The focus on the QTc interval is of clinical importance since significant elongation or an increase greater than 60 ms from baseline are known risk factors for the development of life-threatening arrhythmias [28].

Due to variations in energy drink doses, the impact of these beverages on the QTc interval varied across studies. Studies that used 12-24 oz energy drinks found no significant changes in the QTc interval or other EKG parameters [13,16]. Experimental studies that used higher doses (≥ 32 oz) recorded some lengthening of the QTc interval; however, the increase from baseline remained below established risk parameters [11,12]. Despite this trend with beverage volume, direct comparisons of the EKG are difficult due to variations in intervention, energy drink content, consumption rate, and follow-up time.

In addition to the QTc interval, a T-wave-related parameter has been proposed to be a superior marker for arrhythmia development [12,13]. This parameter is the time difference between the peak of the T-wave and its endpoint (Tp-e interval). It represents the transmural dispersion of ventricular repolarization, and an increase in it is associated with malignant ventricular arrhythmias [29].

Atrial and ventricular arrhythmias have been recorded in case reports. The circumstances leading to arrhythmia development greatly varied across studies, with some subjects solely consuming energy drinks and others co-ingesting them with alcohol. Stress, exercise, and heat exposure were also present as confounding factors across cases [22-25]. Emotional stress and alcohol are identified risk factors for AFib [30,31]. Current evidence also suggests that vigorous exercise may be associated with AFib risk [31]. Therefore, it is unclear whether arrhythmia development occurred due to energy drinks alone or the combined effect of additional influences.

A study from 2015 evaluated lifestyle factors that may have arrhythmogenic effects [32]. It was found that those without an arrhythmia had lower stress levels, lower intake of coffee and energy drinks, and higher intake of fruits and vegetables. Furthermore, 72% of those with arrhythmias reported energy drink use compared to 31% of those without arrhythmias. This may indicate a multifactorial onset of arrhythmia development in relation to energy drinks.

Limitations

While this review utilized high-quality studies, certain limitations must be acknowledged. Included articles were restricted to the English language, and no formal quality or risk-of-bias assessment was performed. Many of the included studies contained short follow-up times and small sample sizes, with the largest study containing 60 participants. Participants are also predominantly young across studies, ranging from 10 to 40 years old, limiting this review's findings to other populations. Studies also varied in interventions, including confounding factors such as exercise and the co-ingestion of alcohol. Prospective studies in this review required participants to consume their beverages within a short time frame, varying anywhere from one minute to one hour [12,13,16,17,20]. This is not fully representative of consumer habits, where beverage consumption is typically staggered. Future studies must take this into account while developing study designs. In terms of case reports, potential publication bias and limited studies among healthy individuals restrict the ability to determine direct causation between energy drinks and arrhythmia genesis. Moreover, only one pediatric study was included in this review. Additional research should investigate the cardiac effects of energy drinks on teenagers.

## Conclusions

While there is evidence of some acute EKG changes with energy drink intake, no clinically significant arrhythmias were reported in small prospective studies. Although available evidence demonstrates that one-time use of high-volume energy drinks may prolong the QTc interval, the increased levels that are reported do not exceed known QTc arrhythmia markers. However, arrhythmia risk following energy drink intake cannot be ruled out. Future studies should evaluate repeated long-term use of these beverages.

Case reports related to otherwise healthy individuals indicate a potential relationship between energy drinks and AFib, especially in the presence of additional triggers such as alcohol. Further research should also aim to address the contribution of lifestyle factors to further assess the impact of energy drinks on arrhythmia genesis.
